# Predictors and Sequelae of Postoperative Delirium in a Geriatric Patient Population With Hip Fracture

**DOI:** 10.5435/JAAOSGlobal-D-20-00221

**Published:** 2021-05-14

**Authors:** Monique S. Haynes, Kareme D. Alder, Courtney Toombs, Ikechukwu C. Amakiri, Lee E. Rubin, Jonathan N. Grauer

**Affiliations:** From the Department of Orthopaedics and Rehabilitation, Yale School of Medicine, New Haven, CT (Ms. Haynes, Mr. Alder, Dr. Toombs, Dr. Rubin, and Dr. Grauer), and the Department of Orthopaedics, Geisel School of Medicine at Dartmouth, Hanover, New Hampshire (Mr. Amakiri).

## Abstract

**Introduction:**

Postoperative delirium is common for patients with hip fracture. Predictors of postoperative delirium and its association with preexisting dementia and adverse postoperative outcomes in a geriatric hip fracture population were assessed.

**Methods:**

Patients with hip fracture aged 60 years and older were identified in the 2016 and 2017 National Surgical Quality Improvement Program Procedure Targeted Databases. Independent risk factors of postoperative delirium were identified. Associations with mortality, readmission, and revision surgery were evaluated using moderation and mediation analysis.

**Results:**

Of 18,754 patients with hip fracture, 30.2% had preoperative dementia, 18.8% had postoperative delirium, and 8.3% had both preoperative dementia and postoperative delirium. Independent predictors of postoperative delirium were as follows: older age, male sex, higher American Society of Anesthesiologists score, dependent functional status, nongeneral anesthesia, preoperative diabetes, bleeding disorder, and preoperative dementia. Preoperative dementia and postoperative delirium each had an independent correlation with 30-day mortality (odds ratios = 2.06 and 1.92, respectively, with *P* < 0.001 for both). However, when both were present, those with preoperative dementia and postoperative delirium had an even higher odds of mortality based on moderation analysis (odds ratio = 2.25, *P* < 0.001). Readmissions and reoperations were significantly correlated with postoperative delirium, but not with preoperative dementia. The combination of preoperative dementia and postoperative delirium, however, did have compounding effects. Furthermore, a significant proportion of the total effect of preoperative dementia on mortality and readmission was accounted for by the development of postoperative delirium based on mediation analysis (medeff: 7%, *P* < 0.001 and medeff: 35%, *P* < 0.001).

**Discussion:**

Postoperative delirium is a potentially preventable postoperative adverse outcome that was seen in 18.8% of 18,754 patients with hip fracture. Those with preoperative dementia seem to be a particularly at-risk subpopulation. Quality improvement initiatives to minimize postoperative delirium in this hip fracture population should be considered and optimized.

In the United States alone, approximately 300,000 geriatric patients experience hip fractures annually.^1,2^ Unfortunately, high morbidity and high mortality exist in this group.^3,4^ This warrants close attention, especially because it is estimated that the yearly incidence of hip fractures occurring yearly could climb to 840,000 by 2040, as the population ages.^1^ Postoperative delirium in the elderly has been associated with increased morbidity, mortality, and high rates of other adverse outcomes after hip fracture surgery.^5-7^

A prospective study by Bellelli et al^5^ found that, for geriatric patients with hip fracture, each day of postoperative delirium increased the risk of mortality at 6 months by 17%. Similarly, another prospective study by Holmes and House^6^ found that geriatric patients with hip fracture with a diagnosis of delirium within 5 days of surgery had prolonged hospital length of stay and increased the risk of 6-month mortality. In addition, a retrospective analysis of a Canadian database by Zywiel et al^8^ found that the development of delirium after hip fracture surgery led to markedly increased length of hospital stay and markedly increased episode-of-care costs.

The incidence of postoperative delirium in the hip fracture population has been reported with broadly variable incidence, ranging from 4.0 to 53.3% of patients.^9,10^ Predictors of delirium for orthopaedic surgery include the following: preoperative functional status,^11^ low body mass index (BMI),^12^ fracture occurring indoors,^12,13^ advanced age,^8,12,14^ diabetes,^14^ substance use,^14^ polypharmacy,^14^ low postoperative hematocrit,^11,14^ and history of dementia.^11^

Preoperative dementia is a factor that bears specific consideration for its relationship with postoperative delirium. As a related cognitive dysfunction, it has been shown to be related to the postoperative delirium in many clinical settings.^12,14,15^ Fick et al^15^ conducted a metanalysis to study the prevalence of and outcomes associated with delirium superimposed on dementia in populations aged 65 years and older. Two of the 14 studies included in their analysis focused on geriatric hip fracture populations. The first study, a prospective cohort study by Edlund et al^10^ including 101 patients, found that most patients with postoperative delirium experienced drops in blood pressure or other postoperative complications such as infections. The second study, a randomized control trial by Marcantonio et al^16^ including 126 patients, found that proactive geriatric consultation beginning in the preoperative period reduced the incidence of postoperative delirium.

Studies evaluating risk factors for postoperative delirium have generally been limited in size to patient populations ranging from 10 to 562 patients.^8,9,11,12,14,15,17^ This study was conducted to study predictors of postoperative delirium and its sequelae in a patient population larger than previously assessed for such analysis. By studying a larger population, the potential moderating and mediating effects of delirium on the relationship between preoperative dementia and perioperative outcomes of interest could be assessed.

## Methods

### Database/Study Population

This study used the 2016 and 2017 National Surgical Quality Improvement Program (NSQIP) Procedure Targeted Hip Fracture Databases merged with the corresponding NSQIP Participant Use Data Files. Our Institutional Review Board has granted exemption for all studies that use NSQIP.

NSQIP variables include patient demographics, comorbidities, and 30-day postoperative events including morbidity and mortality for patients who have undergone surgical procedures.^18^ The targeted hip fracture database contains 17 additional variables and data on 9,390 patients from 117 different sites.^19^

All patients in the hip fracture database who were 60 years of age and older were included in this study. This age limitation was implemented to narrow the scope to geriatric patients who had sustained a hip fracture. Patients with preoperative dementia and patients with postoperative delirium were identified according to database coding. Those patients diagnosed with both preoperative and postoperative delirium were not included in regression analyses to allow for analysis of only newly developed delirium after surgery and its relation to preexisting dementia.

### Demographic and Outcome Variables

The NSQIP database includes patient demographic information: age, sex, height, weight, American Society of Anesthesiologists (ASA) score, and functional status. BMI was calculated from height and weight for each patient. Additional data abstracted included diabetes, hypertension, smoking status, chronic obstructive pulmonary disease, congestive heart failure, use of corticosteroids, bleeding disorder, acute renal failure, and preoperative dementia.

The database also provides surgical information. This includes operations done based on common procedural terminology code, anesthesia type used, length of operation, and postoperative length of stay in the hospital. The NSQIP Procedure Targeted Hip Fracture Databases provides information on diagnoses of preoperative and postoperative dementia and delirium. The targeted databases were merged with the general NSQIP databases to form a data set with comprehensive information for patients with hip fracture.

The primary outcome of interest was mortality within 30 days of the principal surgery. Secondary outcomes of interest included return to the hospital (readmission) and return to the operating room (revision surgery) within 30 days of principal procedure.

### Statistical Analysis

Statistical analysis was done using Stata. All figures presented were created with Microsoft Excel and Lucidchart.

### Univariate and Multivariate Analyses

A series of chi square tests was done to characterize the study population by postoperative delirium status and to determine demographic, comorbidity, and procedural characteristics for which those with and without postoperative delirium differed. Significance was adjusted using Bonferroni correction to account for multiple comparisons.

Multivariate logistic regression was then done to determine independent risk factors for postoperative delirium. The model controlled for demographic factors including age, sex, ASA, BMI, and functional status. It also controlled for patient morbidities and surgical characteristics including preoperative dementia, smoking status, corticosteroid use, bleeding disorder, chronic obstructive pulmonary disease, renal failure, diabetes, hypertension, congestive heart failure, dyspnea, operation time, anesthesia type, and type of procedure performed. Significance was again adjusted using Bonferroni correction to account for multiple comparisons.

### Moderation Analysis

**Figure 1:**
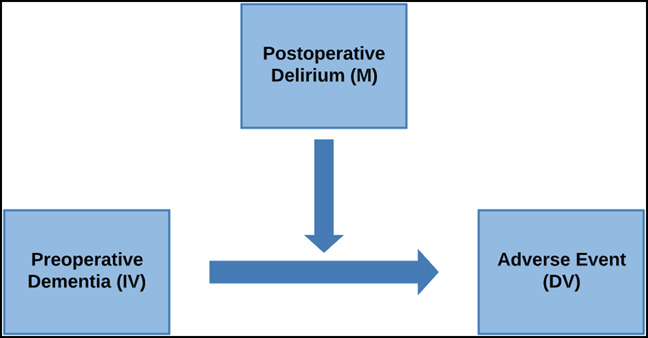
Flow chart showing the moderation model. DV = dependent variable, IV = independent variable, M = moderator

Moderation analysis is also known as an analysis of the interaction between variables.^20^ It is usually done to determine when or in what context the effect of an independent variable (IV) on a dependent variable (DV) occurs or changes.^20^ Considering Figure 1, preoperative dementia is the IV, the adverse event, mortality, for example, is the DV, and postoperative delirium is the moderating variable (M). M is the factor that changes the magnitude of the effect of preoperative dementia on the adverse event. In this case, preoperative dementia directly affects the occurrence of the adverse event, but the interaction of preoperative dementia and postoperative delirium has the ability to modify the size of the effect.

Three logistic regression models were created to determine the moderating effects of postoperative delirium on the relationship between preoperative dementia and (1) mortality, (2) readmission, and (3) revision surgery. Each model controlled for all the aforementioned variables in the independent risk factor model and also contained an interaction term relating preoperative dementia and postoperative delirium. Significance was determined using a *P* value of 0.05.

### Mediation Analysis

**Figure 2:**
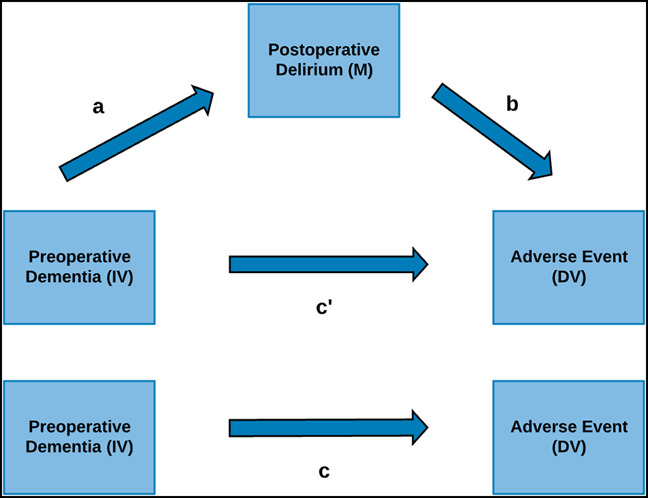
Flow chart showing the mediation model. DV = dependent variable, IV = independent variable, M = mediator. ^a^Effect of DV on M, ^b^effect of M on DV, ^c^'total effect of IV on DV, and ^c^direct effect of IV on DV

Mediation analysis is commonly done to study how an outcome occurs by measuring the total effect of the IV on the DV, the direct effect of the IV on the DV, and the indirect effect of the IV on the DV through a mediating variable (M) that lies on the causal pathway between the IV and the DV.^20,21^ Considering Figure 2, preoperative dementia is the IV, the adverse event is the DV, and postoperative delirium is M. The variable c' represents the direct effect of preoperative dementia on the adverse event. The indirect effect of preoperative dementia on the adverse event occurs through postoperative delirium and is represented by ab. The variable c represents the total effect of preoperative dementia on the adverse event and is equal to ab + c.' The goal of the analysis is to determine the proportion of the total effect that occurs because of the indirect pathway through postoperative delirium.

Following this mediation model, analysis was done using a generalized version of the Erickson method.^22^ The Hicks mediation package was used for confirmation.^23^ The mediating effect of postoperative delirium on the relationship between preoperative dementia and (1) mortality, (2) readmission, and (3) revision surgery was determined.

## Results

### Study Population

**Figure 3:**
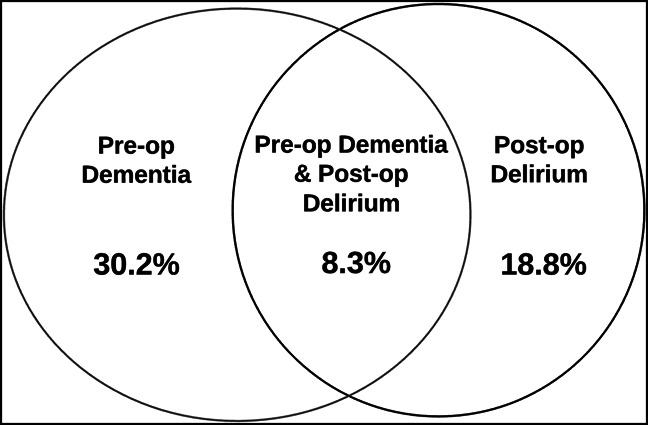
Venn diagram showing the incidences of dementia and postoperative delirium

In total, 18,754 patients with hip fracture 60 years of age and older were identified. Of the total sample, 30.2% had preoperative dementia, 18.8% had a new diagnosis of delirium postoperatively, and 8.3% had both preoperative dementia and postoperative delirium (Figure 3). Of the 5659 patients who had preoperative dementia and 27.3% developed postoperative delirium.

### Postoperative Delirium Associations

Based on univariate analysis, the cohorts with and without postoperative delirium differed significantly based on age (*P* < 0.001), sex (*P* = 0.004), ASA (*P* < 0.001), functional status (*P* < 0.001), anesthesia type (*P* < 0.001), operation time (*P* = 0.011), and postoperative length of stay (*P* < 0.001) (Table 1). They also differed significantly based on several comorbidities: hypertension (*P* < 0.001), CHF (*P* = 0.001), bleeding disorder (*P* < 0.001), and preoperative dementia (*P* < 0.001) (Table 2).

**Table 1:** Demographics by Delirium Status

Characteristic	No Post-op Delirium (N = 15,235)	Post-op Delirium (N = 3,519)	*P* Value
Age			**<0.001**
60-69	**2,010 (13.19)**	**208 (5.91)**	
70-79	**3,548 (23.29)**	**602 (17.11)**	
80+	**9,677 (63.52)**	**2,709 (76.98)**	
Sex			**0.004**
Male	**4,451 (29.22)**	**1,115 (31.69)**	
Female	**10,784 (70.78)**	**2,404 (68.31)**	
BMI			0.008
≤24 kg/m^2^	6,775 (44.47)	1,621 (46.06)	
25-29 kg/m^2^	4019 (26.38)	863 (24.52)	
30-34 kg/m^2^	1,582 (10.38)	326 (9.26)	
≥35 kg/m^2^	2,859 (18.77)	709 (20.15)	
ASA			**<0.001**
≤2	**2,510 (16.48)**	**328 (9.32)**	
≥3	**12,725 (83.52)**	**3,191 (90.68)**	
Functional status			**<0.001**
Independent	**12,116 (79.53)**	**2,448 (69.57)**	
Partially/completely dependent	**3,119 (20.47)**	**1,071 (30.43)**	
Surgical characteristics			0.131
CPT			
Hemiarthroplasty	5,518 (36.22)	1,327 (37.71)	
Plate/screw fixation	1,996 (13.10)	426 (12.11)	
Cephalomedullary nail fixation	7,721 (50.68)	1,766 (50.18)	
Anesthesia			**<0.001**
General	**10,517 (69.69)**	**2,327 (66.13)**	
Other	**4,618 (30.31)**	**1,192 (33.87)**	
Operation time	**63.94 ± 40.82**	**62.04 ± 35.07**	**0.011**
Postoperative length of stay	**5.46 ± 5.60**	**7.50 ± 7.70**	**<0.001**

ASA = American Society of Anesthesiologists, BMI = body mass index, CPT = common procedural terminology

Bolded values represent significance at *P* < 0.006 according to Bonferroni correction.

**Table 2:** Comorbidities by Delirium Status

Comorbidity	No Post-op Delirium (N = 15,235)	Post-op Delirium (N = 3,519)	*P* Value
Diabetes			0.010
Not diabetic	12,548 (82.36)	2,891 (82.15)	
Noninsulin dependent	1,631 (10.71)	340 (9.66)	
Insulin dependent	1,056 (6.93)	288 (8.18)	
Hypertension	**10,258 (67.33)**	**2,482 (70.53)**	**<0.001**
Dyspnea			0.643
No dyspnea	14,081 (92.43)	3,242 (92.13)	
Moderate exertion	1,010 (6.63)	238 (6.76)	
At rest	144 (0.95)	39 (1.11)	
Smoking status	1,548 (10.16)	303 (8.61)	0.005
COPD	1,596 (10.48)	408 (11.59)	0.053
Congestive heart failure	**550 (3.61)**	**168 (4.77)**	**0.001**
Use of corticosteroids	886 (5.82)	191 (5.43)	0.373
Bleeding disorder	**2,508 (16.46)**	**723 (20.55)**	**<0.001**
Acute renal failure	72 (0.47)	22 (0.63)	0.248
Pre-op dementia	**4,112 (26.99)**	**1,547 (43.96)**	**<0.001**

Bolded values represent significance at *P* < 0.005 according to Bonferroni correction.

Based on multivariate regression analysis, age, sex, ASA, functional status, anesthesia type, diabetic status, bleeding disorder, and preoperative dementia were independent risk factors for postoperative delirium. Odds ratios and confidence intervals are presented in Table 3.

**Table 3:** Independent Risk Factors for Post-op Delirium

Characteristic/Comorbidity	OR	99.4% CI	Multivariate *P* Value
Age			**<0.001**
70-79	**1.50**	**1.18-1.91**	
≥80	**2.22**	**1.77-2.79**	
Sex			**<0.001**
Female	**0.85**	**0.76-0.96**	
ASA			**<0.001**
≥3	**1.40**	**1.17-1.67**	
Functional status			**<0.001**
Partially/completely dependent	**1.18**	**1.04-1.35**	
Anesthesia			**<0.001**
Other	**1.18**	**1.05-1.32**	
Diabetes			**<0.001**
Insulin dependent	**1.30**	**1.06-1.59**	
Bleeding disorder	**1.27**	**1.11-1.45**	**<0.001**
Preoperative dementia	**1.75**	**1.55-1.98**	**<0.001**

ASA = American Society of Anesthesiologists, OR = odds ratio

Bolded values represent significance at *P* < 0.006 according to Bonferroni correction.

The mortality rate was 8.7% among patients who developed delirium postoperatively and only 4.9% among patients who did not develop postoperative delirium (*P* < 0.001). Similarly, increases in the rates of the secondary adverse outcomes, readmission and revision surgery, were found to be significantly increased among patients who developed delirium in the postoperative period. The readmission rate was 12.3% among the cohort of patients who developed delirium after surgery and 7.5% among those who did not (*P* < 0.001). The revision surgery rate was 3.6% among those who developed delirium compared with 2.0% among those who did not develop delirium postoperatively (*P* < 0.001) (Table 4).

**Table 4:** Rates of Adverse Events by Delirium Status

Adverse Event	Rate	*P* Value
Mortality		**<0.001**
No post-op delirium	**4.9%**	
Post-op delirium	**8.7%**	
Readmission		**<0.001**
No post-op delirium	**7.5%**	
Post-op delirium	**12.5%**	
Reoperation		**<0.001**
No post-op delirium	**2.0%**	
Post-op delirium	**3.6%**	

Bolded values represent significance at *P* < 0.05.

### Moderation Analysis

Moderation analysis was done to assess the interaction between preoperative dementia and postoperative delirium on mortality, readmission, and revision surgery (Table 5). Patients with only preexisting dementia were 2.06 times more likely than patients without dementia to die after hip fracture surgery (OR = 2.06, 95% CI, 1.57 to 2.35, *P* < 0.001) (Table 5). Those without preexisting dementia who developed postoperative delirium were 1.92 times more likely to die after surgery compared with those without postoperative delirium (OR = 1.92, 95% CI, 1.74 to 2.43, *P* < 0.001) (Table 5). Patients with postoperative delirium superimposed on preexisting dementia were 2.23 times more likely to die after hip fracture repair compared with those without dementia or delirium (OR = 2.25, 95% CI, 1.82 to 2.79, *P* < 0.001) (Table 5).

**Table 5:** Moderating Effects of Post-op Delirium

Adverse Event	Odds Ratio	95% CI	*P* Value
Mortality			
No pre-op dementia	**Reference**	—	—
Pre-op dementia	**2.06**	**1.57-2.35**	**<0.001**
No post-op delirium	**Reference**	—	—
Post-op delirium	**1.92**	**1.74-2.43**	**<0.001**
No pre-op dementia or post-op delirium	**Reference**	—	—
Pre-op dementia + post-op delirium	**2.25**	**1.82-2.79**	**<0.001**
Readmission			
No pre-op dementia	Reference	—	—
Pre-op dementia	1.10	0.95-1.28	0.193
No post-op delirium	**Reference**	—	—
Post-op delirium	**1.57**	**1.34-1.84**	**<0.001**
No pre-op dementia or post-op delirium	**Reference**	—	—
Pre-op dementia + post-op delirium	**1.75**	**1.47-2.10**	**<0.001**
Reoperation			
No pre-op dementia	Reference	—	—
Pre-op dementia	0.88	0.66-1.18	0.396
No post-op delirium	**Reference**	—	—
Post-op delirium	**1.78**	**1.36-2.34**	**<0.001**
No pre-op dementia or post-op delirium	**Reference**	—	—
Pre-op dementia + post-op delirium	**1.74**	**1.25-2.41**	**0.001**

Bolded values represent significance at *P* < 0.05.

Readmission and revision surgery followed a similar pattern. Patients with only preexisting dementia were not significantly more likely to be readmitted or to have revision surgery than those without preexisting dementia (Table 5). However, those without preexisting dementia who developed delirium postoperatively were significantly more likely to be readmitted or to have revision surgery compared with those who did not develop postoperative delirium (Table 5). Patients with postoperative delirium superimposed on preoperative dementia were also significantly more likely to be readmitted or to have revision surgery than those without dementia or delirium (Table 5).

### Mediation Analysis

Mediation analysis was done to assess the indirect effect of postoperative delirium on the relationship between preoperative dementia and mortality, readmission, and revision surgery (Table 6). The analysis determined that postoperative delirium was a significant mediator of the relationship between preoperative dementia and (1) mortality and (2) readmission.

**Table 6:** Mediating Effects of Post-op Delirium

Adverse Event	Mediation Effect	*P* Value
Mortality		
Pre-op dementia + post-op delirium	7%	<0.001
Readmission		
Pre-op dementia + post-op delirium	35%	0.031

Significance determined at *P* < 0.05.

The indirect effect of preoperative dementia on mortality through postoperative delirium accounted for 7% of the total effect of preoperative dementia on mortality (medeff = 7%, *P* < 0.001) (Table 6). In addition, the indirect effect of preoperative dementia on readmission through postoperative delirium accounted for 35% of the total effect of preoperative dementia on readmission (medeff: 35%, *P* < 0.001) (Table 6). Postoperative delirium was not a significant mediator of the relationship between preoperative dementia and revision surgery (*P* > 0.05) (Table 6).

## Discussion

As the population ages, the incidence of hip fractures is expected to increase.^24,25^ Delirium is common after hip fracture surgery^9,10^ and has been associated with adverse outcomes such as death,^16,26^ functional decline,^27^ cognitive decline,^27^ longer hospitalization,^28^ and higher healthcare costs.^29,30^

This study recapitulates both the incidence of and the deleterious effects of delirium in the postoperative patient with hip fracture. Of the 18,235 patients included in the study, 18.8% developed delirium in the postoperative period, which is congruent with Edlund et al^10^ who determined the incidence of delirium in the postoperative patient with hip fracture to be 18.8%.

This study also determined that mortality was increased among patients who developed delirium postoperatively (8.7% vs 4.9%, *P* < 0.001), which is concordant with established literature**.^16,26^ In addition, the study's secondary adverse outcomes, readmission and revision surgery, were found to be increased among patients who developed delirium postoperatively (12.5% vs 7.5% and 3.6% vs 2.0%, respectively and both *P* < 001). Although these results are consistent with the literature,^31^ to our knowledge, this is the first study to evaluate moderation and mediation effects of postoperative delirium on the relationship between preexisting dementia and the occurrence of adverse postoperative events.

Preoperative dementia was present in 30.2% of the study population of which 27.3% developed postoperative delirium. Moderation analysis was done to determine whether the development of postoperative delirium affected the likelihood of mortality, readmission, and revision surgery among patients with preoperative dementia. Mediation analysis was used to study how the development of postoperative delirium affected postoperative outcomes. It determined the proportion of the total effect of preoperative dementia on each outcome of interest that could be accounted for by the development of postoperative delirium. The moderation analysis first showed that postoperative delirium significantly amplified the effects of preoperative dementia on mortality, readmission, and revision surgery (Figure 1). The mediation analysis demonstrated that a significant proportion of the total effect of preexisting dementia on mortality and readmission occurred through postoperative delirium (Figure 2).

Given the negative ramifications of untreated delirium, particularly among patients with hip fracture with preexisting dementia, identifying patients at risk for the development of delirium is paramount. This study's multivariate regression analysis suggests that older age, male sex, higher ASA score, partially/completely dependent functional status, nongeneral anesthesia, insulin-dependent diabetic status, preexisting bleeding disorders, and preoperative dementia are independent risk factors for the development of postoperative delirium in patients with hip fracture (Table 3). Our determination of age,^32,33,34,35^ ASA score^36-38^, functional status,^33,34^ bleeding disorder,^39^ and preoperative dementia^33-35^ are congruent with existing literature. Interestingly, our study found that although most of the study sample was female (70.3%) and most of those who had preexisting dementia were female (73.0%), male patients were markedly more likely to develop delirium after hip fracture repair. Therefore, based on this study's findings and preestablished literature, these independent risk factors may be useful in the identification of patients who are at risk for developing delirium postoperatively and who are therefore at increased risk of experiencing adverse postoperative events.

This study does have limitations. The recognition of delirium and dementia can be challenging, but the NSQIP database does adhere to strict definitions of these conditions. Furthermore, other adverse outcomes and function measures aside from mortality, readmission, and revision surgery could not be studied in the current analyses because of parameters of the database.

In conclusion, hip fracture repair can be associated with preexisting dementia and postoperative delirium. The results of this study demonstrate that postoperative delirium is a moderating variable and markedly increases the risk of postoperative mortality, readmission, and revision surgery, particularly among patients with baseline dementia. In addition, mediation analysis demonstrates that delirium indirectly accounts for a notable proportion of the relationship between preoperative dementia, mortality, and readmission. Therefore, special attention should be given to patients who present with cognitive impairment and other risk factors for postoperative delirium at the time of surgery.
